# Substance use disorder bridge clinics: models, evidence, and future directions

**DOI:** 10.1186/s13722-023-00365-2

**Published:** 2023-04-14

**Authors:** Jessica L. Taylor, Sarah E. Wakeman, Alexander Y. Walley, Laura G. Kehoe

**Affiliations:** 1 Section of General Internal Medicine, Department of Medicine, Boston University School of Medicine and Boston Medical Center, 801 Massachusetts Avenue, Second Floor, Boston, MA 02118 USA; 2 Grayken Center for Addiction, Boston Medical Center, Boston, MA USA; 3 Division of General Internal Medicine, Department of Medicine, Massachusetts General Hospital, Boston, MA USA; 4 Department of Medicine, Harvard Medical School, Boston, MA USA

**Keywords:** Opioid use disorder, Bridge clinic, Buprenorphine, Methadone, Addiction

## Abstract

**Background:**

The opioid overdose and polysubstance use crises have led to the development of low-barrier, transitional substance use disorder (SUD) treatment models, including bridge clinics. Bridge clinics offer immediate access to medications for opioid use disorder (MOUD) and other SUD treatment and are increasingly numerous. However, given relatively recent implementation, the clinical impact of bridge clinics is not well described.

**Methods:**

In this narrative review, we describe existing bridge clinic models, services provided, and unique characteristics, highlighting how bridge clinics fill critical gaps in the SUD care continuum. We discuss available evidence for bridge clinic effectiveness in care delivery, including retention in SUD care. We also highlight gaps in available data.

**Results:**

The first era of bridge clinic implementation has yielded diverse models united in the mission to lower barriers to SUD treatment entry, and preliminary data indicate success in patient-centered program design, MOUD initiation, MOUD retention, and SUD care innovation. However, data on effectiveness in linking to long-term care are limited.

**Conclusions:**

Bridge clinics represent a critical innovation, offering on-demand access to MOUD and other services. Evaluating the effectiveness of bridge clinics in linking patients to long-term care settings remains an important research priority; however, available data show promising rates of treatment initiation and retention, potentially the most important metric amidst an increasingly dangerous drug supply.

## Introduction

Overdose mortality and other medical complications, including hepatitis C virus (HCV), HIV, and bacterial infections, continue to surge among people who use opioids and other criminalized drugs in the United States (US) [1]. Potent fentanyl now dominates the opioid supply in many regions of the country. Over 100,000 people died of drug overdose in the 12-month period ending in December 2021, with over 70,000 of these deaths involving synthetic opioids other than methadone (i.e., fentanyl) [2]. Rates of viral hepatitis, HIV and emergency department (ED) visits and hospitalizations for serious substance use disorder (SUD)-related infections have also climbed [3–8].

Effective treatments for SUD are associated with myriad clinical benefits. For example, buprenorphine and methadone, the first-line medications for opioid use disorder (MOUD), decrease opioid and all-cause mortality and are associate with decreased injection-related risk behaviors and HIV and HCV acquisition [9, 10]. However, access to evidence-based treatment remains inadequate, with barriers that include insufficient treatment program capacity, long wait times for treatment, stigma, and inadequate support during high-risk touch points and transitions, such as ED and hospital discharge and release from incarceration [11–14].

These complex, multi-level challenges have spurred interest in the creation of new, low-barrier and transitional models for SUD care delivery [15, 16]. Also known as low-threshold models, low-barrier SUD treatment programs are characterized by immediate same-day access to SUD treatment (e.g., on a walk-in basis), abbreviated intake processes, trusting relationships between patients and staff, flexibility, and a focus on harm reduction and patients’ individual goals, even if they do not include abstinence [15–21].

Bridge clinics offer a model for low-barrier, transitional SUD care. Usually based in emergency department or outpatient settings, bridge clinics provide rapid initiation of MOUD and medications for other SUDs, stabilization during high-risk transitions, harm reduction services, and linkage to long-term providers [17, 22–24]. While diverse in structure, operations, and funding sources, which often include a combination of institutional support, public health funding, other grant funding, and clinical revenue, bridge clinics are increasingly recognized as integral components of the care continuum for people with SUD. In addition to addressing unmet clinical needs, bridge clinics offer important learning opportunities for trainees including students, residents and addiction medicine fellows, and they can leverage increased operational flexibility to foster innovation in SUD care delivery [22, 25–27]. However, given the relatively recent implementation of bridge clinic models, the impact of these programs has not been well described.

The goal of this narrative review is to describe bridge clinic models and available evidence for their effectiveness vis-à-vis acceptability to patients, success filling gaps in the SUD care continuum, linkage to long-term care and other evidence of MOUD retention, and effectiveness in addressing other clinical care gaps for patients with SUD. We highlight key gaps in available data and offer future directions for bridge clinic work.

## Methods

Two authors (JLT and LGK) compiled a list of known relevant publications. To identify other bridge clinic-related publications, they searched online data bases of PubMed and Embase.

PubMed search terms included “bridge clinic,” “bridge clinics,” “(“bridge clinic”) AND (“Treatment Outcome” [Mesh] OR Outcome, Treatment OR Patient-Relevant Outcome OR Outcome, Patient-Relevant OR Outcomes, Patient-Relevant OR Patient Relevant Outcome OR Patient-Relevant Outcomes OR Clinical Effectiveness OR Effectiveness, Clinical OR Treatment Effectiveness OR Effectiveness, Treatment OR Rehabilitation Outcome OR Outcome, Rehabilitation OR Treatment Efficacy OR Efficacy, Treatment OR Clinical Efficacy OR Efficacy, Clinical). Embase search term included “bridge clinic,” “‘treatment outcome’/exp OR ‘health care outcome and process assessment’ OR ‘outcome and process assessment (health care)’ OR’ outcome and process assessment, health care’ or ‘outcome management’ OR ‘patient outcome’ OR ‘therapeutic outcome’ OR ‘therapy outcome’ or ‘treatment outcome’” as well as a combination of above terms.

JLT and LGK reviewed identified papers and abstracts for relationship to SUD and relevance to the bridge clinic definition, a low-barrier, transitional SUD care setting, outlined above.

In addition, the public-facing websites of published bridge clinic models were reviewed to identify operations details (e.g., hours of operation) presented in Table 1. Publication corresponding authors and/or bridge clinic medical directors were contacted via email to confirm the accuracy of the data included in Table 1.

**Table 1:** Published bridge clinic models, examples, United States, 2023

Outpatient, hospital-based								
Program	Schedule	Setting	Clinical staffing	MOUD	Naloxone	Harm reduction	ID services	Other services
**Faster Paths** Boston Medical Center Boston, MA	7 days 8A-4:30P Scheduled and walk-in access	Primarily in person Audio-only and audio-visual telemedicine visits available	MD or NP RN care manager Patient care coordinators × 2	Methadone, 72-h rule Buprenorphine, SL Buprenorphine, SQ Naltrexone, PO Naltrexone, IM	Prescribed Dispensed	Distribution of safer consumption supplies including needles, safer snorting and smoking materials, & fentanyl test strips Condom distribution	HIV testing (phlebotomy) HIV testing (rapid POCT) HIV PEP & PrEP HIV treatment HCV testing & treatment STI testing STI treatment (on-site) Vaccines	Medications for AUD, nicotine UD, and stimulant UD (off-label); wound care, incision & drainage; linkage to recovery coaching; outpatient alcohol & benzodiazepine withdrawal management; patient supplies; contraception & EC; referral to PCP and behavioral health
**MGH Bridge Clinic** Massachusetts General Hospital Boston, MA	M-F 9A-5P Scheduled and walk-in access	Primarily in person Audio-only and audio-visual telemedicine visits available	MD (internal medicine & psychiatry) Psychologist RN Medical Assistant Social worker Recovery Coach Resource Specialist Patient Service Coordinators × 2 Practice Manager	Buprenorphine, SL Buprenorphine, SQ Naltrexone, PO Naltrexone, IM	Prescribed Dispensed	Distribution of safer consupmtion supplies including needles, safer smoking materials, & fentanyl test strips Condom distribution	HIV testing (phlebotomy) HIV PEP & PrEP HCV testing STI testing STI treatment (on site) Vaccines	Medications for AUD, nicotine UD, and stimulant UD (off-label); wound care, incision & drainage; referral to HIV/HCV treatment; outpatient alcohol withdrawal management; patient supplies; contraception & EC; individual and group support; referral to PCP
**Brigham and Women's Bridge Clinic** Brigham and Women's Hospital Boston, MA	M-F 9A-5P Scheduled and walk-in access		MD (internal medicine & addiction psychiatry) Recovery coaches Social workers Resource specialists Medical assistants	Buprenorphine, SL Buprenorphine, SQ Naltrexone, PO Naltrexone, IM	Prescribed		OPAT Other treatment of deep tissue infections related to injection HCV treatment HIV treatment	Addiction psychiatry care; Care for pregnant people; support navigating Department of Children and Families involvement; individual and group peer support
**Harm Reduction & BRidges to Care (HRBR)** Oregon Health Sciences University Portland, OR	M-F 10A-7P	Primarily virtual	MD or NP Care transitions coordinator × 2 Peer recovery specialist	Buprenorphine, SL Naltrexone	Prescribed Dispensed	Safer consumption supplies	HIV testing (phlebotomy) HIV PEP & PrEP HCV testing STI testing STI treatment	Medications for AUD, nicotine UD, and stimulant UD (off-label); referral to HCV treatment; referral to PCP

Outpatient, Health Center-Based								
Program	Schedule	Setting	Clinical Staffing	MOUD	Naloxone	Harm reduction	ID services	Other services
**Zuckerberg San Francisco General Family Health Center Bridge Clinic ** University of California San Francisco San Francisco, CA	M-F 1P-5P In-person and tele M, W, F. Tele only Tu, Th.	Primarily in person and audio-only telemedicine Audio-visual telemedicine visits available	MD (including trainees from addiciton medicine fellowship and family medicine residency) Substance use navigator RN's Medical assistants Eligibility workers (shared with primary care program)	Buprenorphine, SL Buprenorphine, SQ Naltrexone, PO Naltrexone, IM	Prescribed Dispensed	Distribution of safer consumption supplies including smoking kits (e.g., including bubbles, foil, straight pipes and accompanying supplies) and injection kits	HIV testing (phlebotomy) HIV PEP & PrEP HCV testing & linkage to treatment STI testing STI treatment (on site) Vaccines HIV PEP & PrEP HCV testing & linkage to treatment STI testing STI treatment (on site) Vaccines	Medications for AUD and stimulant use disorder (off-label); wound care; outpatient alcohol & benzodiazepine withdrawal management; smoking cessation; patient supplies; contraception & EC; linkage to primary care, therapy, contingency management, residential treatment, and inpatient detox

Emergency department-based								
Program	Schedule	Setting	Staffing	MOUD	Naloxone	Harm reduction	ID services	Other services
**CA Bridge** Emergency Departments, Multiple Institutions California	24/7	Integrated into emergency department care Some hospitals provide ED-staffed outpatient clinic hours for follow-up	Existing emergency medicine staff Substance use navigator	Buprenorphine, SL	Prescribed Dispensed	Hospital staff offered training on distribution of safer injection equipment, encouraged to partner with local syringe service programs	Hospital staff offered training on HIV and HCV screening	
**Upstate Emergency Opioid Bridge Clinic** State University of New York Upstate Medical University Syracuse, NY	Follow-up clinic open twice per week	Patients started on buprenorphine in the ED are seen by ED providers in an outpatient follow-up clinic. Patients can also be referred by non-ED providers	Emergency medicine providers Peer specialists	Buprenorphine, SL	Prescribed	Safer consumption supplies	Referral to community and hospital departments	Medications for other UD; wound care; referral to PCP and other community resources

Virtual Only								
Program	Schedule	Setting	Staffing	MOUD	Naloxone	Harm reduction	ID services	Other Services
**UPMC Medical Toxicology Telemedicine Bridge Clinic** University of Pittsburgh Medical Center Pittsburgh, PA	M-F 9A-5P	Audio only and audio-visual telemedicine visits	Emergency medicine providers	Buprenorphine, SL	Prescribed	Referral to local resources	HIV PrEP Referral to local resources	Motivational interviewing; medications for AUD; outpatient alcohol & benzodiazepine withdrawal management; referral to local SUD provider
**NYC Health + Hospitals Virtual Buprenorphine Clinic** New York, NY	M-F 9A-5P	Audio only and audio-visual telemedicine visits	Addicition medicine and addiction psychiatry providers	Buprenorphine, SL	Prescribed Home delivery			Referral to psychiatry & behavioral health; on site pharmacy; no cost medications if uninsured; referral to PCP; mobie phone application support; insurance enrollment
**Rhode Island Buprenorphine Hotline**	24/7	Audio only	Addiction medicine, emergency medicine, internal medicine, medical toxicology providers	Buprenorphine SL	Prescribed			Community health worker/peer follow-up; services referral; transportation assistance

*Abbreviations:* OTP, opioid treatment program; AUD, alcohol use disorder; UD, use disorder; ID, infectious diseases; SL, sublingual; SQ, subcutaneous; HIV, human immunodeficiency virus; PEP, post-exposure prophylaxis; PrEP, pre-exposure prophylaxis; POCT, point of care test; HCV, hepatitis C virus; STI, sexually transmitted infection; EC, emergency contraception; OPAT, outpatient parenteral antibiotic therapy

## Bridge clinic models

### Hospital-based, outpatient bridge clinics

Many low-barrier SUD bridge clinics are hospital-based, outpatient practices that are generally not licensed as opioid treatment programs (OTP) [17, 22, 23, 25, 28, 29]. Interdisciplinary teams, which may include registered nurse care managers, social workers, psychologists, case managers, patient navigators, peer recovery coaches, pharmacists, addiction medicine physicians, psychiatrists, and nurse practitioners, deliver clinical care and harm reduction services on a scheduled or walk-in basis, with the specific services offered tailored to program size and staffing (Table 1) [22, 23, 28]. Providers may be trained in internal medicine, family medicine, emergency medicine, and/or psychiatry depending on the model [22, 23, 28, 30, 31]. Although some bridge clinics offer integrated behavioral health services or referral, engagement in psychosocial treatment is not required to access MOUD.

There is no defined length of treatment in the bridge model and the duration of care episodes can vary. For example, patients seen in a bridge clinic in Boston, MA between 2014 and 2019 had a median of 5 total bridge clinic visits (IQR: 2–15) spanning a mean “length of stay” of 36 days (IQR: 1–184), but 1 in 4 patients were followed for 6 months or more [22]. Bridge clinics may offer services for set periods of time before linkage to long-term care, may offer a consult and return model for primary care and other settings, and may, in some circumstances, continue to provide care long-term when patients experience barriers to transition to more traditional care settings [17, 23, 30].

Hospital-based settings offer specific advantages, including facilitation of warm handoffs from ED and inpatient teams. Short wait times to first bridge clinic appointment have been associated with appointment attendance [32]. Co-location with institutional phlebotomy, insurance support, and outpatient pharmacy services also facilitates rapid initiation of medications and infectious disease testing. Hospital-clinic licensure and resources like secure medication dispensing cabinets facilitate administration of scheduled medications like methadone for opioid withdrawal management under the “72-h rule” [24]. Furthermore, hospital-based program may have the resources to stock and later bill more costly injectables like extended-release buprenorphine, enabling rapid medication initiation [27].

However, the need to enter a medical center environment to access bridge clinic services may deter some patients, including those who have experienced past stigma or trauma in healthcare settings, and traditional clinical environments may limit the degree to which programs can eliminate barriers to entry.

In addition to MOUD, bridge clinics generally offer pharmacotherapy for other types of SUD, such as naltrexone and acamprosate for alcohol use disorder, and some bridge clinics incorporate HIV, viral hepatitis, and bacterial sexually transmitted infection (STI) screening, treatment, and prevention services like HIV pre- and post-exposure prophylaxis (PrEP, PEP) to address unmet need [23, 33]. On-site access to vaccines and commonly used antibiotics for STI treatment facilitates ready delivery of these services at the point of care. Some bridge clinics also tailor HIV screening to the needs of their patient populations, offering, for example, both phlebotomy-based and rapid point of care HIV testing using fingerstick blood samples for patients who decline phlebotomy due to difficult venous access and other reasons [34]. Bridge clinics with subspecialty infectious disease expertise have been able to offer expanded services to people who inject drugs (PWID) with complex injection-related infections. For example, a bridge clinic in Boston, MA is an interdisciplinary model that includes an infectious disease physician and offers outpatient parenteral antibiotic therapy (OPAT) and SUD follow-up to stably housed PWID who require prolonged intravenous antibiotic therapy for serious injection-associated infections [35].

### Emergency medicine-based bridge models

Emergency Medicine (EM) based bridge models address the specific needs of patients seeking acute care for OUD and SUD-related complications. A study implemented from March 2019–July 2020 evaluated low-threshold buprenorphine initiation and navigation to outpatient treatment anchored in California EDs within hospital systems prepared to expand buprenorphine across care settings [36]. A total of 52 diverse hospitals enrolled, with 60% of the 12,009 OUD encounters resulting in buprenorphine administration and 40% of patients attending one or more outpatient follow-up visits [36].

A separate EM-based model utilizes addiction-boarded EM physicians to both initiate buprenorphine in the ED and follow patients longitudinally in an outpatient addiction clinic, which they also staff [30, 37].

### Virtual bridge clinics

The COVID-19 pandemic ushered in a series of regulatory changes related to the public health emergency that enabled the initiation of buprenorphine by telemedicine, including by audio-only and audiovisual platforms, without the usual requirement for an initial in-person visit. This led to the creation of both new, exclusively virtual bridge clinic models as well as new telemedicine-based buprenorphine initiation pathways within existing bridge clinics that did not previously offer telehealth [38]. Like outpatient bridge clinics, the goal of virtual bridge clinics was to provide treatment initiation and linkage to a provider for maintenance treatment, though the bridge clinics “may follow patients over several visits until follow-up care is established” [38].

Among described virtual-only bridge models, a Pittsburgh, PA Department of Emergency Medicine developed a virtual bridge clinic offering audiovisual and audio-only consultation for patients with SUD, the majority of whom had OUD [39]. Audio only visits (159/200, 79%) were more common than audiovisual [39].

New, virtual-only bridge clinics were also developed in New York, NY, staffed by Addiction Medicine and Addiction Psychiatry, and in Rhode Island, staffed by Addiction Medicine, Emergency Medicine, Internal Medicine, and Medical Toxicology providers [38]. Like telehealth buprenorphine initiation pathways developed in existing bridge clinics, these models sought to serve those at high risk of opioid overdose death in the context of the COVID-19 pandemic, including people experiencing homelessness and those recently released from incarceration [26, 29, 38]. These two exclusively virtual bridge clinics served a total of 124 patients from March to June, 2020, the majority of whom were publicly insured, seeking buprenorphine initiation, and utilized audio-only visit technology [38].

Existing bridge clinics have also described new, virtual pathways to buprenorphine initiation [26, 29, 38]. For example, a bridge clinic in Portland, OR transitioned to over 90% virtual visits in the week following the Drug Enforcement Agency announcement permitting telemedicine initiation of buprenorphine and continued to operate on a predominantly virtual model, with a significant portion of audio only visits, through August 2020 [28]. Like other programs, this bridge clinic retained in-person visit access for those who lacked the resources to participate in telemedicine [28, 40].

While the shift to telemedicine potentially offered a pathway to lower barriers to MOUD, it also threatened to worsen access for people with limited resources, including those without phones and people experiencing homelessness. A bridge clinic in Boston, MA addressed these barriers by partnering with street outreach teams who could link people in the community to buprenorphine prescribers by audio/video platforms in real time [26].

### Other bridge clinic models

Bridge clinics co-located with inpatient medically managed withdrawal programs have also been described to meet the needs of patients treated with buprenorphine during their inpatient medically managed withdrawal care who do not have post-discharge outpatient buprenorphine care immediately available [41].

### Harm reduction service integration

Many bridge clinics operate in a harm reduction model, prioritizing engagement and flexibility, gaining trust, and partnering with patients to reduce the negative consequences of substance use if their goals do not include abstinence [17, 21]. Bridge clinics provide overdose education and naloxone distribution and, increasingly, other harm reduction services, including on-site distribution of safer injection and safer smoking materials [17, 23, 40].

In addition, bridge clinics also offer low-barrier STI, viral hepatitis, and HIV screening, treatment, and prevention, including point-of-care rapid HIV testing, PrEP, and PEP [33]. Some clinics also offer an array of wrap-around clinical services ranging from emergency contraception, long-acting reversible and injectable contraception, wound care, and interim primary care to support engagement and address unmet needs.

## Evidence

Bridge clinics were borne from the need to provide rapid, on-demand services to patients with SUD, followed by stabilization, and ultimate linkage to long-term care. In reviewing the evidence for this model, we focused on the acceptability of bridge clinics to patients, effectiveness in addressing gaps in the SUD care continuum, linkage to long-term care and other evidence of MOUD retention, and effectiveness in addressing other clinical care gaps for patients with SUD.

### The bridge clinic model is acceptable to patients

Qualitative work with 29 bridge clinic patients in Boston, MA interviewed in January–June 2018 indicates that the transitional, low-threshold model for SUD care is acceptable and desired [17]. Patients specifically identified a welcoming clinical environment, the ability to be seen flexibly including without an appointment, incorporation of harm reduction principles, access to knowledgeable providers who approach substance use with compassion and non-judgement, and support linking to ongoing care, including from peer recovery coaches as contributors to a positive experience in the bridge clinic setting [17].

### Bridge clinics fill gaps in care continuum for patients with SUD

Available data also demonstrate that bridge clinics fill important gaps in the care continuum for patients with SUD, including long wait times for outpatient treatment. An analysis of 657 new patient appointments scheduled at a bridge clinic that provided same- and next-day appointments in Boston, MA over a 12-month period indicated that 47% were scheduled same day, 23% were scheduled next-day, and 30% were scheduled 2 or more days away [32]. This analysis excluded patients referred from the inpatient Addiction Consult Service who had already been initiated on MOUD, thus likely reflecting access for patients seeking to initiate MOUD for the first time. Patients who scheduled same-day (OR 6.9, 95% CI 4.6–10.4) or next-day (OR 1.7, 95% CI 1.1–2.7) appointments were more likely to be seen than those who scheduled 2 or more days away [32], emphasizing the importance of the same-day, on-demand access that bridge clinics provide.

Bridge clinics may also facilitate the feasibility of ED buprenorphine initiation, even during off hours. Patients discharged from the ED outside of bridge clinic hours can be provided with a buprenorphine take home kit or prescription and have short-interval follow-up in the bridge clinic [37].

A 2022 program description of ED-initiated buprenorphine describes a hospital system’s interventions that support linkage from the ED to an on-site bridge clinic [42]. In this model, in addition to ED providers giving verbal instruction to present to the bridge clinic after discharge, an electronic medical record ambulatory referral order was implemented to generate a list of patients the bridge clinic team could contact if the patient does not present for follow-up. Addressing OUD during ED encounters through either real-time buprenorphine initiation or referral to the bridge clinic was associated with OUD care retention [42].

The transition from inpatient hospitalization to outpatient clinical care is similarly a high-risk time for loss-to follow-up and interruption of care. Among 4,959 patients who received an inpatient addiction consult at an academic medical center in Boston, MA from October 2014 to October 2019, 24% received additional services including 9% seen in the same institution’s bridge clinic [22].

### Bridge clinics serve patients with high clinical complexity

Data from bridge clinics indicate that they not only fill gaps in the SUD care continuum but do so for patients with high clinical complexity and significant structural barriers to care, including high rates of fentanyl and multiple substance use, psychiatric and medical comorbidities, homelessness, and acute care utilization [22, 23, 29].

For example, among 1197 patients seen in a hospital-based bridge clinic 2014–2019 in Boston, MA, 83% had OUD. Other prevalent use disorders included alcohol (45%), cocaine (40%), and sedative/benzodiazepine (21%). Overall, 60% had two or more concurrent use disorders [22]. In a separate bridge clinic in Boston, MA, among 142 patients seeking methadone emergency withdrawal management with OTP linkage, 85% tested positive for fentanyl, 66% for both an opioid and a stimulant, and the mean number of substances positive in urine drug screen was 3.0 [24]. Among 393 new patients at the same program, inclusive of all SUDs, two-thirds reported injection drug use and over half had experienced opioid overdose [23].

Comorbid mood disorders and serious mental illness, including anxiety (65%), depression (57%), and schizophrenia (14%) are prevalent among bridge clinic patients [22]. New patients presenting to SUD bridge clinics also have high rates of both known and incident HIV, viral hepatitis, and STI [23]. For example, over 1 in 7 patients seeking methadone emergency withdrawal management in a bridge clinic in Boston, MA has baseline HIV infection, an HIV prevalence almost threefold higher than seen among patients receiving buprenorphine in the same institution’s office-based addiction treatment program over a 12-year period [24, 43].

Approximately 1 in 3 patients in some bridge clinic settings experience homelessness, and a greater proportion (54%) experience housing insecurity [22, 23]. Rates of past-12 month acute care utilization, including ED visits (mean 4.3 per person) and inpatient hospitalizations (mean 0.5 per person), are high in bridge clinic patients, even in analyses limited to care episodes at a single institution [24]. Among 1197 patients (mean age 37 years) treated in a bridge clinic in Boston, MA from 2014 to 2019, known mortality at the end of the 5-year study period was 2% [22].

### Linkage to long-term care and MOUD retention

Our literature review identified limited published data on the outcome of linkage from bridge clinics to long-term care settings. A bridge clinic in Boston, MA described rates of OTP linkage and 1-month retention for patients treated with methadone under 72-h-rule, an underutilized regulation that allows non-OTP providers to administer schedule II medications for opioid withdrawal while linking patients to ongoing care [44]. This protocol creates a pathway for same-day, on-demand methadone opioid withdrawal management with rapid OTP referral, thereby shortening wait times for treatment. Evaluation of the bridge clinic’s first 150 encounters demonstrated high rates of OTP linkage (87%), defined as attending at least one OTP visit for methadone dosing, as well as high rates of 1-month OTP retention (58%) rates [24, 25].

In a cohort of patients referred from an ED to an on-site bridge clinic who attended an initial visit, 56% continued buprenorphine 2 years later based on state prescription monitoring program data [30]. Although data are not presented on the location of the most recent buprenorphine prescription, authors indicate that bridge clinic care is “generally only 8–12 weeks duration,” thus it is inferred that those continuing buprenorphine 2 years after initial bridge clinic visit were successfully linked to long-term care [30].

A 2022 study evaluating predictors of engagement and retention in a Boston, MA bridge clinic also looked at transfer to ongoing care as a secondary outcome [45]. This study found that overall, 70% (1911/2730) of bridge clinic episodes of care resulted in engagement, defined as 2 or more completed visits within an episode of care. Transfer to care rates were notably lower, with 28% of episodes of care ending with a documented transfer to another care setting [45].

The relative lack of published data on linkage may be due to the inherent challenges in data sharing in a model that relies upon referring to other long-term care settings within or outside of the bridge clinic medical system. In addition, it remains unclear what the ideal duration of treatment is in this novel model of transitional care as some patients require or prefer the support offered in a bridge clinic longer term [17]. However, existing data suggest bridge clinics may lead to retention on MOUD and in ongoing SUD care. MOUD retention, regardless of care setting, may ultimately be the best measure of bridge clinic success despite the transitional goal.

Additional data on medication retention include outcomes from 1197 unique patients seen in a hospital-based bridge clinic in Boston, MA from 2014 to 2019, including 867 treated with buprenorphine for a total of 1071 buprenorphine initiations after an initial bridge clinic encounter [22]. Bridge clinic patients continued uninterrupted buprenorphine for a mean of 140 days, though the location of ongoing care—in the bridge clinic or in other long-term programs or primary care that document in the same EHR—was not specified [22]. Naltrexone was initiated 209 times after an initial bridge clinic encounter, primarily for the treatment of alcohol use disorder, and the mean length of uninterrupted naltrexone treatment was 33 days. Patients who received extended-release injectable naltrexone received a mean of 1.18 injections [22].

In a convenience sample of 40 serial adults who received one or more extended-release buprenorphine injections in a Boston, MA bridge clinic between February 2019 and July 2019, only 14 discontinued the injectable formulation during the study period, including 10 who switched back to sublingual buprenorphine [27].

Among patients with OUD treated in a virtual bridge clinic during the COVID-19 pandemic, the overwhelming majority (185/192, 96%) filled at least one buprenorphine prescription in the first 30 days after the bridge clinic visit and most (147/192, 77%) filled at least 2 buprenorphine prescriptions. The rate of buprenorphine prescribing and proportion of patients filling at least one and two or more buprenorphine prescriptions did not vary by visit type (i.e., audio only vs. audiovisual) [39]. In two other virtual bridge clinic models, over 80% of patients had a follow-up visit within 30 days [38].

The outcomes addressed in these papers indicate that MOUD initiation and retention are a primary focus of bridge clinic teams, and ongoing research is needed to further examine linkage to care rates.

### Impact on other SUD treatment outcomes

The impact of bridge clinic care compared to usual care on other SUD treatment outcomes, including non-prescribed opioid use, opioid overdose, and injection-related infections, and acute care utilization remains under study. A randomized controlled trial comparing bridge clinic referral to treatment as usual among hospitalized patients with OUD initiating buprenorphine/naloxone or naltrexone began recruitment in 2019 [31].

However, early descriptive data indicate potential positive impacts on ongoing substance use and acute care utilization. For example, a case series of 40 serial patients treated with extended-release buprenorphine in a bridge clinic demonstrated that 65% had toxicology results that were negative for non-prescribed opioid use [27].

Additionally, among 379 patients with OUD referred from an ED in New York State to an on-site bridge clinic, 269 (71%) attended their bridge clinic appointment. In this cohort of 269 patients completing a bridge clinic visit, the volume of ED visits in the six months after their first bridge clinic visit (n = 381) was 42% lower than the volume of ED visits (n = 654) in the six months before their first bridge clinic visit [30]. Reductions in ED utilization were seen among patients with both high and low rates of pre-bridge clinic ED utilization [30].

### Delivery of infection screening and treatment services

As discussed above, several bridge clinic models integrate infection screening, prevention, and treatment services. Foundational bridge clinic work has identified unmet infection-related needs in the bridge clinic population, including high rates of both baseline and newly diagnosed HIV, HBV, and HCV and high rates of gonorrhea, chlamydia, and syphilis infection [23]. Opportunities for vaccination due to non-immunity to HBV (37.8%) and HAV (43.9%) were also high [23].

Data on infection treatment in the same cohort indicated that 88% of bacterial STI were treated and over 1 in 3 patients with newly diagnosed chronic viral infection was linked to ongoing care [23]. Though comparable to other care settings, low HCV treatment linkage rates prompted the bridge clinic to begin to offer on-site, integrated HCV treatment.

Integrated OPAT and SUD follow-up in a bridge clinic has also been associated with high rates of antibiotic course completion. In a retrospective study of 20 stably housed PWID treated in this model in an interdisciplinary bridge clinic in Boston, MA with an infectious diseases physician, 100% completed their antibiotic course [35].

Finally, bridge clinic teams have identified ways in which national HIV PrEP guidelines fall short and highlighted the need for improved access to HIV rapid testing to reduce new HIV infections among PWID [33, 34, 40]. Bridge clinic patients have high rates of eligibility for HIV PEP and PrEP, and work at a bridge clinic in Boston, MA has demonstrated that PEP/PrEP delivery in a bridge clinic is feasible, though opportunities to standardize HIV risk assessments and increase PEP/PrEP uptake remain [33].

## Discussion

As the opioid and multiple substance use crises continue to drive overdose deaths, injection related morbidity, hospital costs, and unmeasurable pain and suffering, bridge clinics have emerged to provide rapid access to MOUD and linkage to long-term SUD care. Early data indicate that the bridge clinic model is acceptable to patients, addresses well-described gaps in the SUD continuum of care, and is effective in the delivery of MOUD and other clinical services tailored to the needs of people who use drugs. However, published data on effectiveness of the bridge clinic model in linking to long-term care—an original goal of the model—are limited.

Available bridge clinic models share an emphasis on low-barrier and immediate medication initiation and transitional care, linking to ongoing community-based care after stabilization, sometimes in a “hub and spoke” or “consult and return” model. Most models employ some degree of harm reduction, ranging from overdose prevention and naloxone distribution to same-day MOUD and provision of safer injection and smoking materials.

Bridge clinics vary in the SUD medications provided, with some providing only buprenorphine treatment for OUD, while others also provide interim methadone for opioid withdrawal management and linkage to OTP, and medication for other SUDs. Some have medication on site to dispense. Some incorporate SUD and psychiatric dual diagnosis care, optional behavioral health support, or interim primary and infectious disease care including hepatitis C and HIV care, PrEP, and PEP, STI treatment, vaccination, and even OPAT for serious injection-associated infections.

The transitional nature of bridge clinics is closely related to their ability to maintain on-demand, walk-in access, presumably via connecting patients to other long-term care settings. Indeed, the need for interval settings that “bridge” patients to long term care was the impetus for this model.

While the high OTP linkage (87%) and 1-month retention (58%) rates among bridge clinic patients treated with methadone under the 72-h rule provides proof of concept and a randomized controlled trial of bridge clinic care is ongoing, additional peer reviewed data are needed on the rates of successful linkage to long term care among bridge clinic patients on buprenorphine [24, 31]. This finding should serve as a call to action for bridge clinic teams to publish programmatic data on linkage outcomes.

Furthermore, the ability to link bridge clinic patients to long term care settings depends on relationships with community programs that are willing and able to meet patients’ ongoing needs. Because low-barrier and harm-reduction focused programs remain relatively rare in traditional outpatient settings, and bridge clinic patients may wish to continue care in the bridge clinic setting long-term, it is not clear that referral is the right outcome for all patients [17, 21]. Bridge clinics caring for patients who require ongoing low barrier drop-in care due to severe SUD with chaotic use, lack of housing, or psychiatric illness must balance individual patient needs with the access-related implications of providing longitudinal care. Consideration towards resourcing bridge clinics to serve as a primary source of care for people with complex SUD is warranted given bridge clinics’ unique ability to support and engage those at very high risk of morbidity over time.

Relatedly, data on high rates of co-morbid serious mental illness indicate that bridge clinics have increasingly become a rapid entry point for people with SUD and severe psychiatric illness unable to access care, many of whom need comprehensive services not readily available in existing bridge clinic models. Data on the psychiatric needs of bridge clinic patients are essential to determine the optimal staffing and approach, be it access to on-demand transitional psychiatric care within the bridge clinic, in collaborating departments, or rapid linkage to long-term psychiatric care. While bridge clinic models will continue to vary by health system and setting, additional data on the optimal staffing model, treatment intensity, and treatment duration for high-touch, on-demand SUD bridge clinics would benefit organizations seeking to launch or sustain bridge clinics.

Cost analyses are also needed to understand and document the beneficial impact of bridge clinics on ED utilization, inpatient admission, and overall cost of care. Evaluations of the cost effectiveness of specific roles and staffing ratios would facilitate the implementation of high value programs even in lower resource settings. Furthermore, although preliminary data indicate positive impacts on SUD treatment access, MOUD initiation, MOUD retention, and receipt of other high-value services, additional data clinical outcomes including linkage to long-term care settings, retention in SUD care, overdose, and mortality, are needed including via randomized trials.

Bridge clinics benefit health systems by serving as sites of innovation in SUD care delivery as well as educational hubs and models for other care settings [23, 24, 26–28, 42]. Many bridge clinics are located within or connected to academic medical centers, making them optimal sites for educational experiences to teach current and future SUD providers and teams. Furthermore, bridge clinics’ flexible staffing and on-demand access, often with the capacity for daily in person visits, offers a unique setting in which to pilot higher-touch clinical interventions. Bridge clinics have rapidly responded to the need for new and innovative pathways to MOUD, offering low-dose buprenorphine induction, rapid initiation of extended-release injectable buprenorphine, and on-demand methadone opioid withdrawal management with OTP linkage, in addition to expanding harm reduction services to support patients facing an increasingly dangerous drug supply [17, 24, 27, 46, 47]. Bridge clinics developed and launched telemedicine for buprenorphine initiation during the COVID-19 pandemic, including flexible approaches responsive to the specific needs of people experiencing homelessness who often lack phones, such as partnering with street outreach [26, 28, 29, 38]. These experiences could offer lessons for other types of care delivery. Bridge clinics have also responded to local HIV outbreaks among PWID by increasing HIV testing and prevention services [40]. In so doing, these small and flexible programs have continued to drive ongoing efforts to de-stigmatize and incorporate evidence-based, patient-centered SUD care into general medical settings.

This narrative review should be viewed with several limitations. The majority of published literature on bridge clinics comes from a handful of academic medical centers, raising the possibility of publication bias. Furthermore, many institutions that have published bridge clinic data are located in large urban areas with comprehensive networks of SUD treatment services and robust state Medicaid coverage, so findings may not generalize to settings with fewer services or more complex insurance barriers. It is not clear from available data whether bridge clinics have been effective in centering racial equity in order to combat increasing racial and ethnic inequities in overdose mortality. Future work should focus on the ideal components of the bridge clinic model, evaluating clinical outcomes through an equity lens, developing access to low-barrier long-term care settings for linkage, and identifying the financial value of the model.

Overall, bridge clinics represent a promising and substantive innovation designed to address the challenges patients have long faced in accessing SUD care in traditional settings and the morbidity and mortality associated with these delays. This first era of bridge clinic implementation has yielded diverse models united in the mission to lower barriers to SUD treatment entry, and preliminary data indicate success in patient-centered program design, MOUD initiation, and SUD care innovation. Whether bridge clinics are effective in linking patients to long-term care settings has not been definitively answered by published data. However, amidst a drug supply substantially more dangerous than when bridge clinics launched, MOUD retention in any setting—including through continued bridge clinic care for those patients unwilling or unable to transfer to traditional outpatient programs—is a key benchmark for overdose reduction and engagement in treatment. As bridge clinics continue to evolve, tools for identifying those patients who can be rapidly transferred after stabilization and those likely to require long-term care in a bridge clinic setting will be helpful in ensuring bridge clinics maintain the on-demand access central to their mission.

## Data Availability

Not applicable.
